# Radiation treatment patterns for breast cancer brain metastases: an NCDB analysis

**DOI:** 10.1007/s10549-026-07955-z

**Published:** 2026-04-22

**Authors:** Thamilini Pathmarajah, Yevgeniya Gokun, Nicole Williams, Gina M. Sizemore, Heather Lefebvre, Margaret E. Gatti-Mays, Brandon Slover, Sierra Daniel, Tanun Jitwatcharakomol, Jacob Eckstein, Therese Andraos, Rebekah Young, John Grecula, Raju Raval, Raj Singh, Simeng Zhu, Dukajin Blakaj, Arnab Chakravarti, Joshua Palmer, Daniel Stover, Sachin R. Jhawar, Sasha Beyer

**Affiliations:** 1 Department of Radiation Oncology, The Ohio State University Comprehensive Cancer Center, Columbus, OH USA; 2 Center for Biostatistics, The Ohio State University Wexner Medical Center, Columbus, OH USA; 3 Division of Medical Oncology, Department of Internal Medicine, The Ohio State University Comprehensive Cancer Center, Columbus, OH USA

**Keywords:** Breast cancer, Brain metastases, Stereotactic radiation, Whole brain radiation, Metastatic breast cancer

## Abstract

**Purpose:**

Breast cancer (BC) brain metastases (BM) treatment involves radiotherapy (RT), surgery, and CNS-penetrating systemic therapies. This study evaluated treatment patterns in brain RT and corresponding survival outcomes among patients with BC BM using the National Cancer Database (NCDB).

**Methods:**

Patients diagnosed with BC BM between 2010 and 2021 were identified. RT was categorized as whole brain (WBRT) vs. stereotactic (SRT). We fitted Overlap Propensity Score Weighting (OPSW) Cox models to account for confounders affecting OS. Variables included age, race, ethnicity, Charlson–Deyo score, insurance, molecular subtype, facility type, and systemic therapy.

**Results:**

Of 8909 patients with BC BM, 43.4% received brain RT (74.1% WBRT, 25.9% SRT). Patients that are African American, lower income, urban, triple-negative, or at community facilities were more likely to receive WBRT over SRT (*p* < 0.05). Median OS for the entire cohort was 10.9 months (95% CI 10.4–11.5). Systemic therapy alone (HR 0.40, 95% CI 0.36–0.43) or combined with RT (HR 0.38, 95% CI 0.35–0.42) improved OS; however RT alone did not improve survival on MVA (HR 0.96 (95% CI 0.91–1.02). Among RT recipients, SRT was associated with improved OS vs. WBRT (HR 0.76, 95% CI 0.69–0.83). Older age, comorbidities, lack of insurance, community facilities, and aggressive subtypes were associated with worse OS.

**Conclusions:**

Treatment patterns, particularly access to SRT, differ among BC BM patients therefore highlighting the need for strategies to promote equitable implementation of evidence-based guidelines. More prospective trials are also needed to establish evidence-based treatment standards for BC BM.

**Supplementary Information:**

The online version contains supplementary material available at 10.1007/s10549-026-07955-z.

## Introduction

Patients with breast cancer brain metastases (BC BM) are treated with a multidisciplinary approach involving CNS-penetrant systemic therapies in addition to local therapies, including surgery and radiation therapy (RT). The treatment of BC BM has evolved toward more frequent use of stereotactic radiotherapy (SRT) due to its excellent local control outcomes and reduced neurocognitive toxicity compared to whole brain radiation therapy (WBRT). Brown et al. reported that among patients with 1–3 brain metastases (8.5% with breast primaries), the use of SRT compared to SRT + WBRT had less cognitive deterioration and no difference in overall survival [1]. More recently, Aizer et al. (2026) reported the Phase III randomized trial showing that stereotactic radiation reduced interference with daily functioning in patients with 5–10 brain metastases (22% with breast primaries) compared to HA-WBRT [2]. The American Society of Clinical Oncology (ASCO)/Society of Neuro-Oncology (SNO)/American Society for Radiation Oncology (ASTRO) guidelines for brain metastases state that SRT may be preferred for patients with better prognosis and those with CNS-penetrant systemic therapy options [3]. Moreover, the NCCN guidelines (Version 3.2025) state that SRT is recommended in patients with “limited” brain metastases based on the number of brain metastases or volume of intracranial disease [4–6] with a definition of “limited” that continues to evolve and depends on the clinical situation [4].

However, previous retrospective studies have shown that sociodemographic variables are also associated with treatment patterns for brain radiation. Kann et al. [7] has previously shown through analysis of NCDB data that non-White race, non-private insurance, lower socioeconomic status, and lower educational attainment were associated with lower use of SRT for brain metastases among patients with primary metastatic non-small cell lung cancer, breast cancer, colorectal cancer, or melanoma. Moreover, Shih et al. [8] showed that while SRT utilization has increased between 2004 and 2020 for brain metastases from > 18 different primaries, there continue to be disparities for patients with lower socioeconomic status, uninsured/public insurance, or non-academic center treatment, potentially reflecting access disparities or differences in disease burden. Additionally, an NCDB analysis by Mainwaring et al. [9] highlighted that patients diagnosed with BCBM between 2004 and 2014 who received SRT differed from those who received WBRT on variables of income, insurance status, and treatment setting.

The current study aimed to identify how sociodemographic factors may impact SRT vs. whole brain radiation therapy treatment patterns for patients with BC BM and better understand the associated survival outcomes in the era of emerging modern CNS-penetrant systemic therapies.

## Methods

### Data source and study cohort

Patients diagnosed with de novo breast cancer brain metastases between 2010 and 2021 were identified in the National Cancer Database (NCDB). The NCDB only captures Breast cancer patients with presence of de novo brain metastases at the time of breast cancer diagnosis (defined per NCDB codebook). Patients with breast cancer and a diagnosis of brain metastases were included. As brain metastases were not recorded by the NCDB until 2010, patients prior to this were excluded. Patients without known follow-up were excluded [10].

### Study variables

Covariates for this analysis included patient age, race, ethnicity, Charlson-Deyo score (CDS), health insurance status, census-derived median income and education for the patient’s zip code, urban/rural residence, treatment facility type, tumor grade, nodal status, molecular subtype, receipt of chemotherapy, immunotherapy, hormone therapy and sites of metastases at the diagnosis (bone, liver and lung). CDS per NCDB codebook is defined as a summary index and is captured at the time of diagnosis with the following categories: 0, 1, 2 or 3 + comorbid conditions. Molecular subtype was defined based on the NCDB variables for estrogen receptor (ER), progesterone receptor (PR) and human epidermal growth factor receptor 2 (HER-2). Systemic therapy was composite indicator of receipt of chemotherapy, immunotherapy and/or hormone therapy.

### Main exposure

**Fig. 1:**
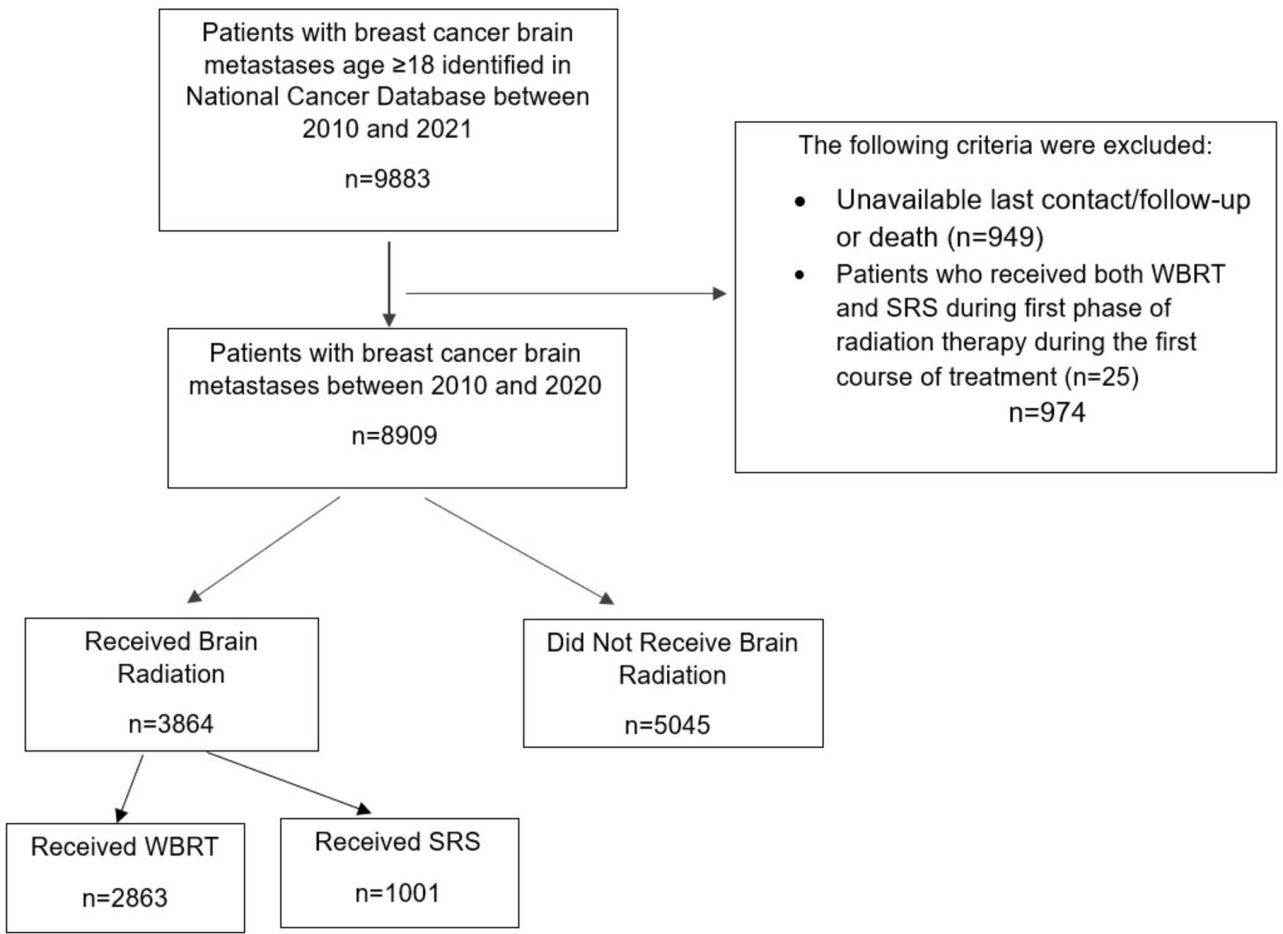
Study schema illustrating selection of patient cohort from NCDB

Brain radiation was defined based on Phase I through III Radiation Primary Treatment Volumes treated during first phase of radiation during first course of treatment. Per NCDB codebook, SRT cohort was defined per the following: “Treatment is directed at one or more sub-sites of the brain but not the whole brain. Chart may describe SRS, Stereotactic Radiosurgery or Gamma Knife”. Also, WBRT cohort is defined per the following: “Treatment is directed at all the brain and its meninges (Whole Brain)”. Both cohorts identify the primary treatment volume or primary anatomic target treated during the first phase of radiation therapy during the first course of treatment. Patients receiving both stereotactic radiation and whole brain radiation during their first course of treatment were excluded from the study (Fig. 1).

### End point

The primary outcome was all-cause mortality. The follow-up period was defined as the number of months from date of initial diagnosis of breast cancer to the event date, defined as (1) date of death, (2) date of the last follow-up, or (3) the end of the study, whichever came first. Patients who did not experience the event were censored at the date of the last follow-up or at the end of the follow-up, whichever occurred earlier.

### Statistical analysis

Descriptive statistics such as medians and interquartile ranges (IQRs) were used for continuous variables while frequencies and proportions were used for categorical variables. Differences between brain RT versus no brain RT groups were compared using Wilcoxon Rank Sum Test for continuous variables and Chi-square or Fisher’s exact test for categorical variables.

We used Overlap Propensity Score Weighting (OPSW) to account for the impact of confounding by indication which results from the differences in baseline characteristics that affect receipt of appropriate group or treatment type [11]. Overlap weighting is a propensity score method that aims to imitate crucial features of randomized clinical trials, such as covariate balance and statistical precision [12]. OPSW has advantageous statistical properties such as exact balance of the means of every covariate included in the logistic regression models used to estimate propensity score.

Propensity scores were estimated using a multivariable binary logistic regression model with group type (brain RT vs no brain RT) as the outcome and age, race, ethnicity, Charlson-Deyo score, insurance, community, subtype, treatment facility type and receipt of systemic therapy (composite measure) as covariates.

The proportionality of hazards assumption was assessed using Schoenfeld residuals. It was violated when comparing patients who received brain radiation therapy vs those who did not receive and OS, hence OPSW Cox regression with robust variance was used to assess the relationship between time-varying interaction between receipt of brain RT and receipt of systemic therapy and overall survival adjusting.

These analyses were repeated for subset of patients who received brain RT, specifically comparing those who received specific brain radiation type (SRT or WBRT). Propensity scores were estimated using a multivariable binary logistic regression model with group type (SRT vs no WBRT) as the outcome and age, race, ethnicity, Charlson–Deyo score, insurance, community, subtype, treatment facility type, chemotherapy, immunotherapy and hormone therapy as covariates. Proportionality was not violated hence OPSW Cox proportional hazard model with robust variance was used to assess the relationship between brain radiation type and OS.

To assess the effectiveness of overlap propensity score weighting, we compared patients between these two therapy groups using standardized differences. Standardized difference is defined as absolute difference in means or proportions divided by pooled standard deviation. A standardized difference of less than 0.1 indicates insignificant imbalance between baseline characteristics and group type [13].

All analyses were performed using SAS version 9.4 (SAS Institute Inc). Two-sided tests were considered statistically significant at a significance level of 0.05.

## Results

### Patient population

8909 patients with BC BM met the study inclusion criteria. Of the total cohort, 43.4% (*n* = 3864) underwent brain RT for BC BM. 74.1% (*n* = 2863) received WBRT, while 25.9% (*n* = 1001) received SRT. Patients who received both SRT and WBRT were excluded (*n* = 25). The study schema for patient selection is provided in Fig. 1.

The median age was 61 years (Interquartile range [IQR] 52–69). 80.1% of patients had a Charlson-Deyo score of 0, indicating a low comorbidity burden. The majority of patients identified as White race (76.6%) and non-Hispanic (92.3%) ethnicity. Median follow-up was 9.8 months.

### Receipt of brain RT vs. no brain RT

Table 1 compares clinical and treatment characteristics among patients with BC BM treated with brain radiation (*n* = 3864) versus those who did not (*n* = 5045). Patients who received RT were significantly younger (median age 60, IQR 52–68 vs. median age 62, IQR 53–70, *p* < 0.001) and had private insurance (40.8% vs 35.3%, *p* < 0.001). There were no significant associations between receipt of brain RT and race (*p* = 0.608), ethnicity (*p* = 0.071), education (*p* = 0.249), community type (*p* = 0.071), or treatment facility type (*p* = 0.448).

**Table 1:** Sociodemographic, clinical and treatment characteristics comparing receipt of brain radiation to no brain radiation among patients with breast cancer brain metastases (*n* = 8909)

	Total (*n* = 8,909)	Brain Radiation (*n* = 3,864)	No Brain Radiation (*n* = 5,045)	*P*-value
Age				< .0001
Median (IQR)	61 (52, 69)	60 (52, 68)	62 (53, 70)	
Race, *n* (%)				0.6084
White	6767 (76.6%)	2946 (76.9%)	3821 (76.4%)	
African American/Black	1638 (18.6%)	714 (18.6%)	924 (18.5%)	
Asian	274 (3.1%)	111 (2.9%)	163 (3.3%)	
Other	151 (1.7%)	60 (1.6%)	91 (1.8%)	
Ethnicity, *n* (%)				0.0708
Non-Hispanic	8011 (92.3%)	3508 (92.9%)	4503 (91.9%)	
Hispanic	667 (7.7%)	268 (7.1%)	399 (8.1%)	
Charlson–Deyo Score, *n* (%)				0.3872
0	7133 (80.1%)	3118 (80.7%)	4015 (79.6%)	
1	1184 (13.3%)	505 (13.1%)	679 (13.5%)	
2	361 (4.1%)	152 (3.9%)	209 (4.1%)	
3 +	231 (2.6%)	89 (2.3%)	142 (2.8%)	
Insurance, *n* (%)				< .0001
Not Insured	642 (7.3%)	261 (6.9%)	381 (7.7%)	
Private Insurance	3294 (37.7%)	1553 (40.8%)	1741 (35.3%)	
Medicaid	1418 (16.2%)	634 (16.6%)	784 (15.9%)	
Medicare	3308 (37.9%)	1334 (35.0%)	1974 (40.1%)	
Other Government	73 (0.8%)	26 (0.7%)	47 (1.0%)	
Median Income Quartiles, *n* (%)				0.0273
< $46,227	1513 (17.0%)	666 (17.2%)	847 (16.8%)	
$46,227–$57,856	1806 (20.3%)	769 (19.9%)	1037 (20.6%)	
$57,587–$74,062	1797 (20.2%)	814 (21.1%)	983 (19.5%)	
$74,063 or higher	2582 (29.0%)	1063 (27.5%)	1519 (30.1%)	
Unknown	1211 (13.6%)	552 (14.3%)	659 (13.1%)	
Education, *n* (%)				0.2487
15.3% or higher	1919 (21.5%)	799 (20.7%)	1120 (22.2%)	
9.1–15.2%	2293 (25.7%)	1018 (26.3%)	1275 (25.3%)	
5.0–9.0%	2129 (23.9%)	911 (23.6%)	1218 (24.1%)	
< 5.0%	1380 (15.5%)	598 (15.5%)	782 (15.5%)	
Unknown	1188 (13.3%)	538 (13.9%)	650 (12.9%)	
Community, *n* (%)				0.0713
Rural	134 (1.5%)	62 (1.7%)	72 (1.5%)	
Urban	1083 (12.5%)	500 (13.4%)	583 (11.8%)	
Metro	7445 (86.0%)	3176 (85.0%)	4269 (86.7%)	
Treatment facility type, *n* (%)				0.4483
Community Cancer Program	594 (7.1%)	239 (6.6%)	355 (7.5%)	
Comprehensive Community Cancer Program	3164 (37.9%)	1377 (38.1%)	1787 (37.8%)	
Academic/Research Program	2873 (34.4%)	1259 (34.8%)	1614 (34.1%)	
Integrated Network Cancer Program	1714 (20.5%)	741 (20.5%)	973 (20.6%)	
Subtype, *n* (%)				<0 .0001
ER+/PR /HER2-	2647 (29.7%)	1012 (26.2%)	1635 (32.4%)	
ER+/PR (+ or −)/HER2 +	1190 (13.4%)	571 (14.8%)	619 (12.3%)	
ER−/PR−/HER2 +	938 (10.5%)	492 (12.7%)	446 (8.8%)	
ER−/PR−/HER2-	1705 (19.1%)	893 (23.1%)	812 (16.1%)	
Unknown	2429 (27.3%)	896 (23.2%)	1533 (30.4%)	
Chemotherapy, *n* (%)				< .0001
Yes	4789 (54.8%)	2485 (65.4%)	2304 (46.6%)	
No	3955 (45.2%)	1317 (34.6%)	2638 (53.4%)	
Hormone therapy, *n* (%)				0.5764
Yes	3440 (39.3%)	1507 (39.7%)	1933 (39.1%)	
No	5303 (60.7%)	2291 (60.3%)	3012 (60.9%)	
Immunotherapy, *n* (%)				< .0001
Yes	1626 (18.4%)	862 (22.4%)	764 (15.2%)	
No	7234 (81.6%)	2983 (77.6%)	4251 (84.8%)	
Systemic therapy, *n* (%)				< .0001
Yes	6516 (73.3%)	3188 (82.6%)	3328 (66.1%)	
No	2379 (26.7%)	672 (17.4%)	1707 (33.9%)	
Bone-Mets at diagnosis, *n* (%)				< .0001
Yes	5850 (66.3%)	2217 (57.9%)	3633 (72.7%)	
No	2978 (33.7%)	1613 (42.1%)	1365 (27.3%)	
Liver-Mets at diagnosis, *n* (%)				0.0002
Yes	2792 (32.0%)	1136 (29.9%)	1656 (33.7%)	
No	5924 (68.0%)	2662 (70.1%)	3262 (66.3%)	
Lung-Mets at diagnosis, *n* (%)				0.0236
Yes	3989 (46.0%)	1789 (47.3%)	2200 (44.9%)	
No	4690 (54.0%)	1990 (52.7%)	2700 (55.1%)	

Patients with triple-negative metastatic breast cancer were more likely to receive brain RT than patients with ER + brain metastases (*p* < 0.001). Moreover, patients with extracranial disease, including the presence of extracranial bone metastases (*p* < 0.001) or liver metastases (*p* < 0.001) were less likely to undergo brain RT. Receipt of systemic therapy (defined as any chemotherapy, immunotherapy or hormone therapy) was significantly associated with receipt of brain RT (82.6% vs 66.1%, *p* < 0.001). However, chemotherapy (*p* < 0.001) and immunotherapy (*p* < 0.001) showed stronger associations with receipt of RT than hormone therapy (*p* = 0.576).

Following OPSW, most sociodemographic and clinical characteristics were well-balanced between those who received vs didn’t receive brain radiation; only a few factors had elevated standardized differences slightly above the 0.1 threshold (Supplemental Table 1).

### Receipt of SRT vs WBRT

Table 2 compares sociodemographic, clinical, and treatment characteristics among patients with breast cancer brain metastases receiving WBRT (*n* = 2863) vs. SRT (*n* = 1001). African American patients (19.4% vs. 16.4%, *p* = 0.013), those with median income < $46,227 (18.2% vs. 14.5%, *p* = 0.007), urban location (14.2% vs. 10.9%, *p* = 0.023), triple-negative subtype (24.2% vs. 20.0%, *p* = 0.051), and comprehensive community cancer treatment facilities (39.2% vs. 34.9%, *p* < 0.001) were more likely to receive WBRT compared to SRT. In contrast, White patients (78.5% vs. 76.3%), those with median income of $74,063 or higher (30.7% vs. 26.4%, *p* = 0.007) and treatment at academic facilities (40.4% vs. 32.9%, *p* < 0.001) were more likely to undergo SRT compared to WBRT (see Tables 3, 4).

**Table 2:** Sociodemographic, clinical and treatment characteristics comparing receipt of whole brain radiation therapy (WBRT) to stereotactic radiosurgery (SRS) among patients with breast cancer brain metastases (*n* = 3864)

	Total (*n* = 3864)	WBRT (*n* = 2863)	SRS (*n* = 1001)	*P*-value
Age				0.0746
Median (IQR)	60 (52, 68)	60 (52, 68)	59 (51, 67)	
Race, *n* (%)				0.0132
White	2946 (76.9%)	2166 (76.3%)	780 (78.5%)	
African American/Black	714 (18.6%)	551 (19.4%)	163 (16.4%)	
Asian	111 (2.9%)	72 (2.5%)	39 (3.9%)	
Other	60 (1.6%)	49 (1.7%)	11 (1.1%)	
Ethnicity, *n* (%)				0.4754
Non-Hispanic	3508 (92.9%)	2596 (93.1%)	912 (92.4%)	
Hispanic	268 (7.1%)	193 (6.9%)	75 (7.6%)	
Charlson–Deyo Score, *n* (%)				0.0840
0	3118 (80.7%)	2293 (80.1%)	825 (82.4%)	
1	505 (13.1%)	391 (13.7%)	114 (11.4%)	
2	152 (3.9%)	107 (3.7%)	45 (4.5%)	
3 +	89 (2.3%)	72 (2.5%)	17 (1.7%)	
Insurance, *n* (%)				0.0907
Not Insured	261 (6.9%)	207 (7.4%)	54 (5.4%)	
Private insurance	1553 (40.8%)	1132 (40.2%)	421 (42.4%)	
Medicaid	634 (16.6%)	453 (16.1%)	181 (18.2%)	
Medicare	1334 (35.0%)	1003 (35.6%)	331 (33.3%)	
Other Government	26 (0.7%)	20 (0.7%)	6 (0.6%)	
Median income quartiles, *n* (%)				0.0066
< $46,227	666 (17.2%)	521 (18.2%)	145 (14.5%)	
$46,227–$57,856	769 (19.9%)	589 (20.6%)	180 (18.0%)	
$57,587–$74,062	814 (21.1%)	593 (20.7%)	221 (22.1%)	
$74,063 or higher	1063 (27.5%)	756 (26.4%)	307 (30.7%)	
Unknown	552 (14.3%)	404 (14.1%)	148 (14.8%)	
Education, *n* (%)				0.0159
15.3% or higher	799 (20.7%)	594 (20.7%)	205 (20.5%)	
9.1–15.2%	1018 (26.3%)	771 (26.9%)	247 (24.7%)	
5.0–9.0%	911 (23.6%)	692 (24.2%)	219 (21.9%)	
< 5.0%	598 (15.5%)	411 (14.4%)	187 (18.7%)	
Unknown	538 (13.9%)	395 (13.8%)	143 (14.3%)	
Community, *n* (%)				0.0230
Rural	62 (1.7%)	43 (1.6%)	19 (2.0%)	
Urban	500 (13.4%)	395 (14.2%)	105 (10.9%)	
Metro	3176 (85.0%)	2335 (84.2%)	841 (87.2%)	
Subtype, *n* (%)				0.0508
ER+/PR+ /HER2−	1012 (26.2%)	724 (25.3%)	288 (28.8%)	
ER+/PR(+ or −)/HER2+	571 (14.8%)	424 (14.8%)	147 (14.7%)	
ER−/PR−/HER2+	492 (12.7%)	365 (12.7%)	127 (12.7%)	
ER−/PR−/HER2−	893 (23.1%)	693 (24.2%)	200 (20.0%)	
Unknown	896 (23.2%)	657 (22.9%)	239 (23.9%)	
Treatment facility type, *n* (%)				< .0001
Community cancer program	239 (6.6%)	196 (7.3%)	43 (4.6%)	
Comprehensive community cancer program	1377 (38.1%)	1054 (39.2%)	323 (34.9%)	
Academic/research program	1259 (34.8%)	885 (32.9%)	374 (40.4%)	
Integrated network cancer program	741 (20.5%)	556 (20.7%)	185 (20.0%)	
Chemotherapy, *n* (%)				< .0001
Yes	2485 (65.4%)	1787 (63.5%)	698 (70.7%)	
No	1317 (34.6%)	1028 (36.5%)	289 (29.3%)	
Hormone therapy, *n* (%)				0.0081
Yes	1507 (39.7%)	1082 (38.4%)	425 (43.2%)	
No	2291 (60.3%)	1733 (61.6%)	558 (56.8%)	
Immunotherapy, *n *(%)				0.0001
Yes	862 (22.4%)	595 (20.9%)	267 (26.8%)	
No	2983 (77.6%)	2252 (79.1%)	731 (73.2%)	
Bone-Mets at diagnosis, *n* (%)				0.0671
Yes	2217 (57.9%)	1669 (58.7%)	548 (55.4%)	
No	1613 (42.1%)	1172 (41.3%)	441 (44.6%)	
Liver-Mets at diagnosis, *n* (%)				0.3428
Yes	1136 (29.9%)	854 (30.3%)	282 (28.7%)	
No	2662 (70.1%)	1962 (69.7%)	700 (71.3%)	
Lung-Mets at diagnosis, *n* (%)				0.0037
Yes	1789 (47.3%)	1366 (48.7%)	423 (43.3%)	
No	1990 (52.7%)	1437 (51.3%)	553 (56.7%)

**Table 3:** Association between receipt of brain radiation therapy for breast cancer brain metastases and overall survival

	Crude HRs (95% CI)	Adjusted HRs (95% CI)*
Age		
1-year increase	1.020 (1.018–1.022)	1.012 (1.009–1.016)
5-year increase	1.10 (1.09–1.11)	1.06 (1.05–1.08)
Race		
White	Ref.	Ref.
African American/Black	1.14 (1.08–1.21)	1.08 (1.00–1.17)
Asian	0.79 (0.68–0.91)	0.82 (0.68–0.98)
Other	0.68 (0.55–0.83)	0.94 (0.73–1.21)
Ethnicity		
Non-Hispanic	Ref.	Ref.
Hispanic	0.65 (0.59–0.72)	0.71 (0.63–0.80)
Charlson–Deyo Score		
0	Ref.	Ref.
1	1.27 (1.19–1.36)	1.17 (1.07–1.27)
2	1.55 (1.38–1.74)	1.23 (1.07–1.42)
3 +	1.74 (1.51–2.00)	1.41 (1.15–1.74)
Insurance		
Not Insured	1.35 (1.23–1.48)	1.35 (1.19–1.53)
Private Insurance	Ref.	Ref.
Medicaid	1.12 (1.05–1.21)	1.13 (1.04–1.24)
Medicare	1.52 (1.44–1.60)	1.06 (0.98–1.15)
Other Government	1.20 (0.91–1.54)	1.17 (0.72–1.88)
Median income quartiles		
< $46,227	1.19 (1.11–1.28)	–
$46,227–$57,856	1.20 (1.12–1.28)	–
$57,587–$74,062	1.19 (1.11–1.27)	–
$74,063 or higher	Ref.	–
Unknown	1.10 (1.02–1.19)	–
Education		
15.3% or higher	1.07 (0.99–1.16)	–
9.1–15.2%	1.21 (1.12–1.31)	–
5.0–9.0%	1.09 (1.01–1.18)	–
< 5.0%	Ref.	–
Unknown	1.07 (0.98–1.17)	–
Community		
Rural	1.20 (0.99–1.43)	1.09 (0.88–1.36)
Urban	1.13 (1.06–1.21)	1.08 (1.00–1.18)
Metro	Ref.	Ref.
Subtype		
ER+/PR +/HER2-	Ref.	Ref.
ER+/PR (+ or −)/HER2 +	0.84 (0.77–0.90)	0.90 (0.82–0.98)
ER−/PR−/HER2 +	1.08 (0.99–1.18)	1.13 (1.02–1.25)
ER−/PR−/HER2−	1.96 (1.83–2.09)	1.84 (1.69–2.00)
Unknown	1.54 (1.45–1.64)	1.32 (1.22–1.42)
Treatment facility type		
Community cancer program	1.26 (1.14–1.39)	1.19 (1.05–1.35)
Comprehensive community cancer program	1.25 (1.18–1.32)	1.21 (1.13–1.30)
Academic/research program	Ref.	Ref.
Integrated network cancer program	1.25 (1.17–1.34)	1.26 (1.17–1.36)

**Table 4:** Association between receipt of brain radiation therapy type for breast cancer brain metastases and overall survival

	Crude HRs (95% CI)	Adjusted HRs (95% CI)*
Age		
1-year increase	1.021 (1.018–1.024)	1.012 (1.006–1.018)
5-year increase	1.11 (1.09–1.13)	1.06 (1.03–1.10)
Race		
White	Ref.	Ref.
African American/Black	1.17 (1.07–1.29)	1.14 (0.98–1.31)
Asian	0.74 (0.60–0.92)	0.69 (0.51–0.95)
Other	0.70 (0.51–0.95)	0.98 (0.56–1.71)
Ethnicity		
Non-Hispanic	Ref.	Ref.
Hispanic	0.62 (0.53–0.73)	0.74 (0.60–0.92)
Charlson–Deyo Score		
0	Ref.	Ref.
1	1.33 (1.20–1.48)	1.18 (1.01–1.37)
2	1.57 (1.30–1.89)	1.33 (1.07–1.65)
3 +	1.85 (1.46–2.35)	1.61 (1.24–2.10)
Insurance		
Not insured	1.32 (1.15–1.52)	1.25 (1.02–1.53)
Private insurance	Ref.	Ref.
Medicaid	1.04 (0.94–1.16)	1.13 (0.99–1.30)
Medicare	1.55 (1.43–1.69)	1.16 (1.01–1.33)
Other government	1.34 (0.83–2.15)	1.11 (0.53–2.34)
Median income quartiles		
< $46,227	1.24 (1.12–1.39)	–
$46,227−$57,856	1.32 (1.18–1.46)	–
$57,587−$74,062	1.25 (1.13–1.38)	–
$74,063 or higher	Ref.	–
Unknown	1.18 (1.06–1.32)	–
Education		
15.3% or higher	1.20 (1.07–1.36)	–
9.1–15.2%	1.23 (1.10–1.38)	–
5.0–9.0%	1.15 (1.03–1.29)	–
< 5.0%	Ref.	–
Unknown	1.16 (1.02–1.32)	–
Community		
Rural	1.18 (0.90–1.55)	1.20 (0.88–1.64)
Urban	1.21 (1.09–1.34)	1.06 (0.91–1.23)
Metro	Ref.	Ref.
Subtype		
ER+/PR +/HER2-	Ref.	Ref.
ER+/PR (+ or −)/HER2+	0.73 (0.66–0.82)	0.96 (0.79–1.17)
ER−/PR−/HER2 +	0.99 (0.88–1.11)	1.08 (0.89–1.32)
ER−/PR−/HER2−	1.81 (1.64–2.01)	1.45 (1.20–1.75)
Unknown	1.37 (1.24–1.51)	1.27 (1.11–1.46)
Treatment facility type, *n* (%)		
Community cancer program	1.36 (1.16–1.60)	1.20 (0.94–1.53)
Comprehensive community cancer program	1.33 (1.22–1.45)	1.24 (1.11–1.38)
Academic/research program	Ref.	Ref.
Integrated network cancer program	1.26 (1.14–1.39)	1.22 (1.07–1.39)
Chemotherapy		
Yes	0.54 (0.50–0.59)	0.58 (0.52–0.65)
No	Ref.	Ref.
Hormone therapy		
Yes	0.67 (0.63–0.72)	0.61 (0.54–0.70)
No	Ref.	Ref.
Immunotherapy		
Yes	0.51 (0.47–0.56)	0.64 (0.56–0.73)
No	Ref.	Ref.
Type of brain radiation		
Whole bran radiation therapy (WBRT)	Ref.	Ref.
Stereotactic radiosurgery (SRS)	0.79 (0.72–0.86)	0.76 (0.69–0.83)

### Receipt of brain RT and associated survival outcomes

The median OS for the entire cohort of patients with breast cancer brain metastases was 10.9 months (95% CI 10.4–11.5), including a 1-year OS rate of 48% (95% CI 47–49%) and a 2-year OS rate of 34% (95% CI 33–35%). The Kaplan–Meier curve in Supplemental Fig. 2 shows that patients treated with brain RT had improved survival compared to those not treated with brain radiation (HR 0.89 (95% CI 0.85–0.93)) within the first 4 years after treatment. After 4 years, brain radiation no longer conveyed significantly improved survival outcomes, most likely due to the limited number of cases with > 4 years survival (Supplemental Table 1). Regardless, the median OS for the RT cohort was 12.7 months (95% CI 11.9–13.6), significantly longer median OS than the entire cohort.

**Fig. 2:**
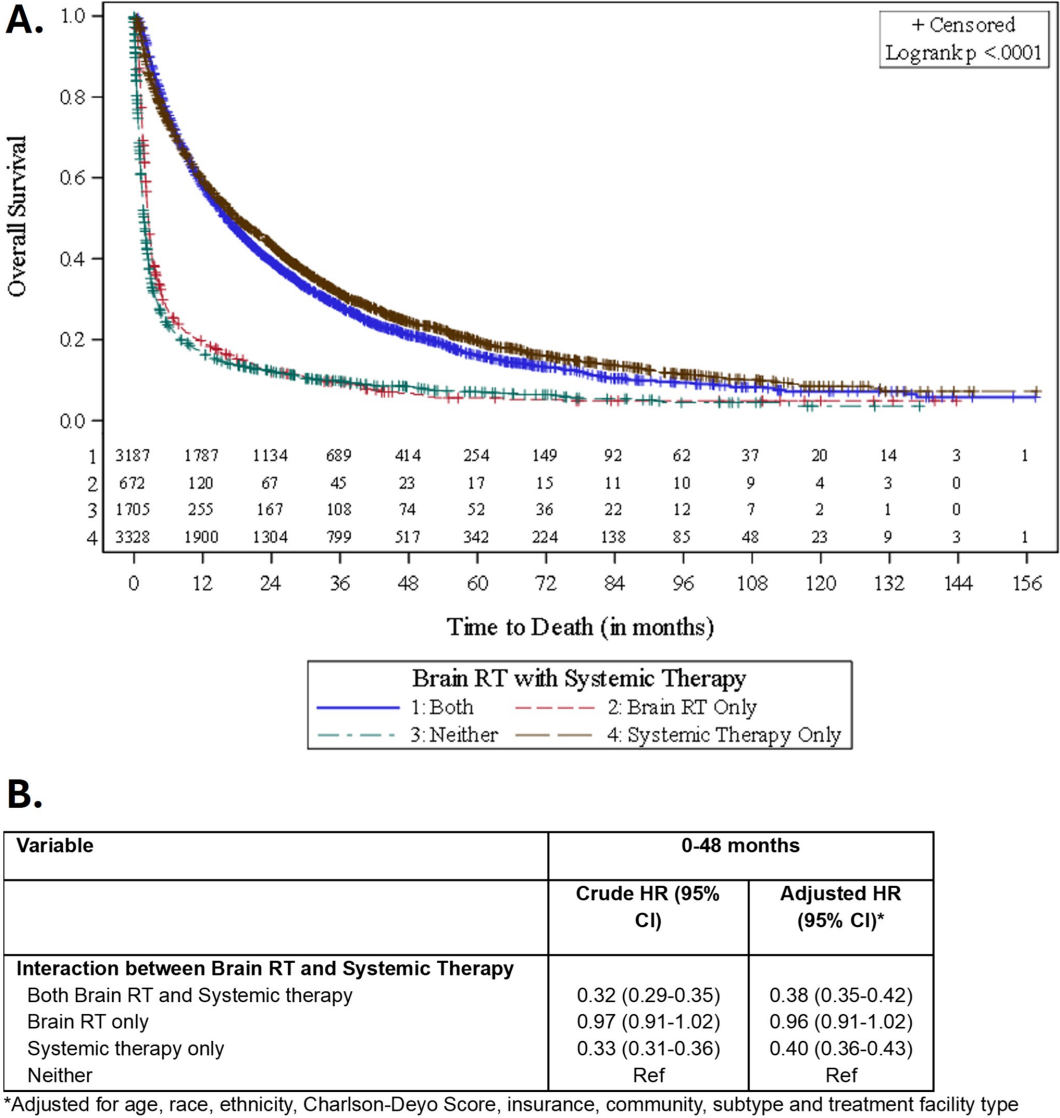
**A** Kaplan–Meier curve comparing OS among patients treated with systemic therapy only, brain RT only, both, or neither for breast cancer brain metastases. **B** Time-varying association between brain radiation receipt and systemic therapy with overall survival

However, in our adjusted model, patients receiving systemic therapy only (HR: 0.40, 95% CI 0.36–0.43) or receipt of both brain RT and systemic therapy (HR: 0.38, 95% CI 0.35–0.42) had significantly better survival outcomes than those receiving brain radiation alone or neither systemic therapy nor brain RT. Patients on systemic therapy alone as well as patients with both brain RT and systemic therapy receipt continued to have better survival, even after 4 years post-diagnosis (Fig. 2).

**Fig. 3:**
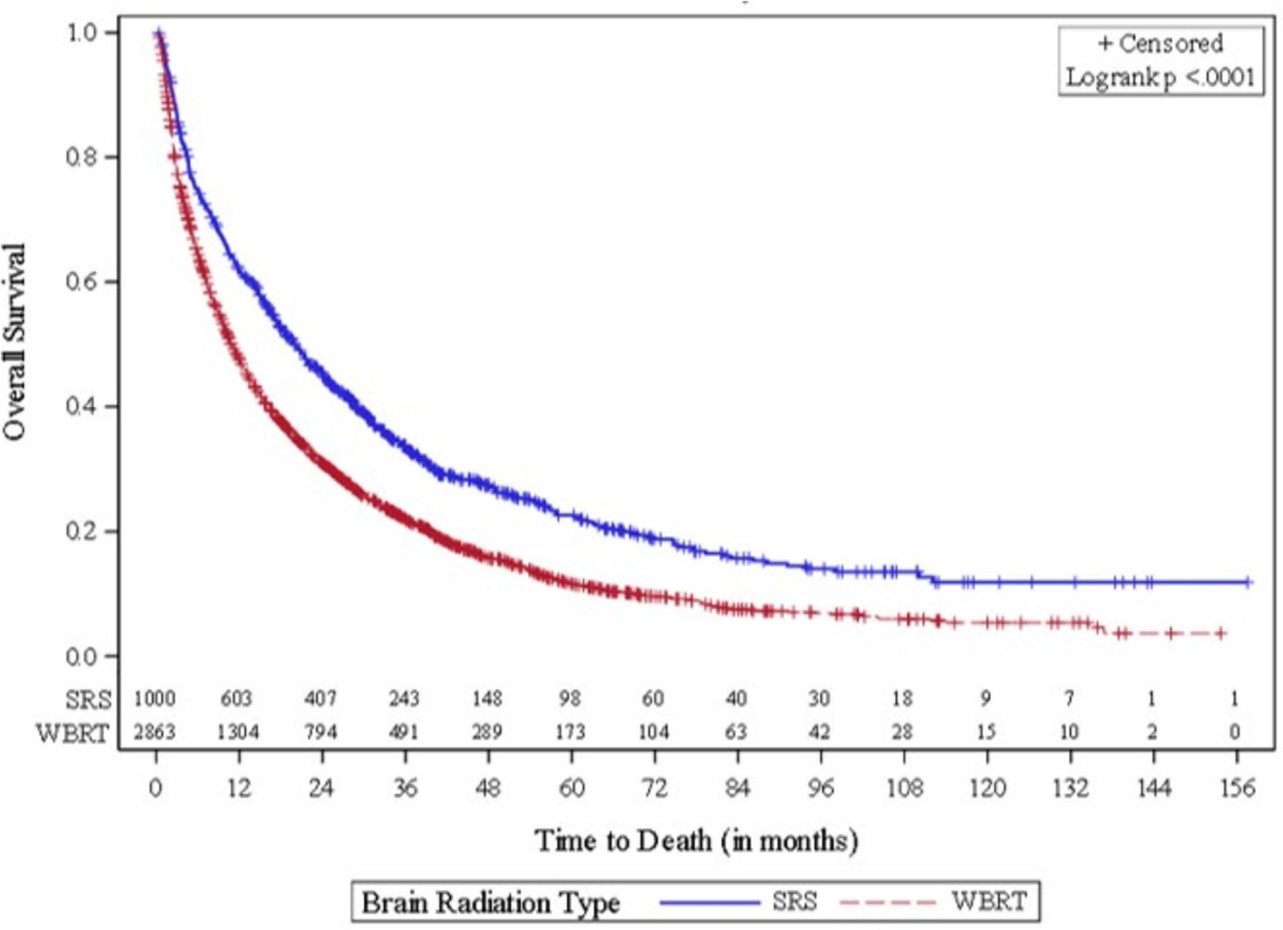
Kaplan–Meier curve modeling OS between patients receiving WBRT or SRT for breast cancer brain metastases

Among the cohort of patients that underwent brain radiation (SRT or WBRT), patients undergoing SRT had better survival outcomes compared to patients undergoing WBRT (HR: 0.76, 95% CI 0.69–0.83) after adjusting for age, race, ethnicity, CDS, insurance, community, subtype, treatment facility type, chemotherapy, hormone therapy and immunotherapy (Fig. 3).

## Discussion

The diagnosis of BC BM remains a devastating event for patients. However, multidisciplinary management—including brain radiation, neurosurgical resection, and increasingly effective CNS-penetrant systemic therapies—has improved survival outcomes while also aiming to minimize treatment-related toxicity. Contemporary guidelines recommend SRT for patients with “limited” brain metastases; however, the definition of limited disease based on number and volume of brain metastases remains variable, and substantial practice variation persists among institutions and providers. This study aimed to better understand whether treatment patterns for SRT vs. WBRT may not only be determined by clinical criteria, but also by sociodemographic characteristics of patients within the National Cancer Database (NCDB).

Our findings are consistent with prior literature demonstrating disparities in the receipt of SRT. African American patients, those residing in lower-income areas, patients treated at community cancer programs, and those living in urban locations were more likely to receive WBRT rather than SRT. In addition, patients with triple-negative disease were more likely to undergo WBRT. These findings align with those reported by Kann et al. [7], whose NCDB analysis demonstrated that non-White race and non-private insurance were associated with lower utilization of SRT among patients with brain metastases across multiple primary tumor types. Similarly, Mainwaring et al. [9] highlighted several structural barriers to SRT access in patients diagnosed between 2004 and 2014, including geographic availability of stereotactic technology, referral patterns, institutional resources, and the availability of multidisciplinary subspecialty expertise. There have also been numerous studies showing that patients with lower socioeconomic status may present with more advanced disease, including intracranial metastases, due to poor health literacy or financial concerns about healthcare costs [14–17]. Therefore, these patient populations may have presented with a larger burden of disease in the brain, therefore contributing to higher rates of WBRT.

Despite the gradual shift toward greater adoption of SRT over the past two decades, WBRT remained more frequently utilized than SRT during the entire study period (Supplemental Fig. 1). Although the proportion of patients treated with SRT increased over time, only approximately one-third of radiation patients diagnosed with BC BM in 2020 underwent SRT (Supplemental Fig. 1). These findings likely reflect persistent structural barriers to SRT implementation. SRT requires specialized equipment, such as Gamma Knife, CyberKnife, or advanced linear accelerator–based platforms, as well as trained multidisciplinary teams and treatment planning expertise that are more concentrated at large academic or tertiary referral centers. Such resources are not uniformly available across treatment centers, particularly in community or rural settings. Additionally, referral pathways, travel distance to tertiary centers, insurance coverage, and other patient barriers may ultimately affect whether patients receive stereotactic treatment.

Interestingly, patients who received SRT in our analysis were associated with a 24% lower risk of death compared with those who received WBRT. This observation differs from randomized trials demonstrating similar overall survival (OS) outcomes between SRT and WBRT. The randomized trial by Brown et al. [1] demonstrated that while SRT alone resulted in significantly less cognitive decline compared with WBRT plus SRT in patients with 1–3 BM (8.5% of which had breast primaries), there was no difference in overall survival. Similarly, Aizer et al. [2] reported comparable survival outcomes between SRT and WBRT in patients with 5–20 brain metastases (22% of which had breast primaries). In contrast, SRT provides excellent local control and improved quality of life, whereas WBRT is associated with improved intracranial progression-free survival without an overall survival advantage.

The observed survival difference in our study is therefore most likely attributable to selection bias inherent to retrospective database analyses. Patients treated with WBRT typically present with a higher intracranial disease burden, poorer performance status, or more widespread extracranial disease—factors that may independently predict for worse overall survival. In contrast, SRT is generally reserved for patients with more limited disease—fewer brain metastases, smaller cumulative tumor volumes, and better functional status. Importantly, the NCDB does not capture key intracranial disease characteristics such as the number and volume of metastases, brain metastasis velocity, or the presence of leptomeningeal disease. As a result, our ability to fully account for treatment selection and survival differences is limited. Furthermore, the NCDB does not include information on symptoms, neurocognitive outcomes or quality of life, which also represent major considerations when selecting between SRT and WBRT.

Patients receiving systemic therapy alone (HR 0.40, 95% CI 0.36–0.43) or both systemic therapy and brain radiation (HR 0.38, 95% CI 0.35–0.42) within 4 years of diagnosis had significantly improved survival compared with patients who received neither systemic therapy nor brain radiation. Moreover, brain radiation alone was not associated with improved overall survival. These findings are consistent with prior work suggesting that systemic therapy is the primary driver of survival outcomes among patients with BC BM. Hajijazi et al. [18], for example, demonstrated that the introduction of HER2-targeted therapies significantly improved survival in patients with HER2-positive brain metastases. Rieke et al. [19] also showed that systemic treatment after BM diagnosis was associated with long-term survival in BC patients with BM from the BMBC registry.

Our study also demonstrated worse outcomes among patients with triple-negative and HER2-positive breast cancer subtypes. These findings likely reflect the historically aggressive biology of these subtypes and their propensity for CNS involvement. Mechanistic insights have been proposed to explain these differences; Cosgrove et al. [20] demonstrated that HER2-positive BCBM exhibit downregulation of metabolic enzymes including ALDOA, GPI, and ENO1, while triple-negative tumors demonstrate reduced activation of CD8+ T-cell–mediated immune pathways, potentially contributing to immune evasion and aggressive intracranial disease behavior.

Importantly, most patients included in this analysis were diagnosed prior to the widespread availability of modern CNS-penetrant systemic therapies and changing landscape on management of breast cancer brain metastases. Since 2020, several agents, including tucatinib, trastuzumab-deruxtecan, Sacituzumab govitecan and datopotumab deruxtecan, have become FDA-approved after demonstrating intracranial activity in metastatic breast cancer. The HER2CLIMB trial demonstrated that the addition of tucatinib to trastuzumab and capecitabine significantly improved overall survival and intracranial progression-free survival in patients with HER2-positive metastatic breast cancer, including those with active brain metastases with an intracranial objective response rate (ORR-IC of 47.3%) [21, 22]. Similarly, the DESTINY-Breast trials established that trastuzumab-deruxtecan as a highly effective therapy for HER2-positive metastatic disease with an ORR-IC of 73.3% [23, 24]. In triple-negative disease, sacituzumab govitecan has also demonstrated improved survival outcomes [25]. These therapies represent a paradigm shift in the management of BC BM and have significantly expanded the role of systemic therapy in controlling intracranial disease.

Consistent with these advances, our study demonstrated a gradual improvement in overall survival among patients with BCBM diagnosed between 2010 and 2021. Survival outcomes for patients with HER2-positive and triple-negative disease will likely continue to improve as more recent NCDB datasets capture patients treated with these newly approved agents. Furthermore, our findings that patients receiving both systemic therapy and brain radiation had the best survival outcomes further support the central role of systemic therapy in determining prognosis.

While the large sample size of the NCDB represents a major strength of this analysis, several limitations must be acknowledged. As a retrospective database study, our analysis is subject to selection bias and unmeasured confounding variables. The NCDB does not provide detailed information regarding the extent of intracranial disease, including the number or volume of metastases, the presence of leptomeningeal disease, or brain metastasis velocity. Similarly, important clinical factors such as patient performance status and burden of extracranial disease are not captured. These factors likely influenced treatment recommendations for SRT versus WBRT. Additionally, the NCDB does not include information regarding intracranial control, recurrence patterns, symptomatic response, or neurocognitive outcomes. Because one of the principal advantages of SRT over WBRT is preservation of neurocognitive function and improved quality of life, the inability to assess these endpoints represents an important limitation. Furthermore, the patient cohort included in this study was diagnosed between 2010 and 2021, meaning that many patients were treated prior to the routine incorporation of modern CNS-penetrating systemic therapies. The NCDB also lacks detailed information regarding specific systemic agents received, making it difficult to determine which patients received therapies with known intracranial activity.

Classifying treatment (brain RT and systemic therapy) as simply “received vs not received” can induce immortal time bias when the timing of treatment initiation is not aligned with the start of follow-up—that is, patients must survive long enough to receive the specific therapy. This issue is well described in the causal inference literature and can materially exaggerate apparent treatment benefits if not addressed. In the NCDB, one key constraint is that the timing fields for systemic and/or radiation therapies are frequently incomplete or discordant with the indicator that treatment occurred, which limits our ability to code both systemic therapy and radiation therapy as time-dependent exposures in a consistent way across all patients. Prior work has documented substantial missingness in NCDB treatment variables, and the NCDB’s own documentation emphasizes variability in data capture across sites and years—factors that complicate precise timing analyses [10]. Given these data limitations, our primary analyses used the available treatment indicators but we attempted to account for timing where feasible. Specifically, for brain radiation, exploratory analyses (based on KM curve in Supplemental Fig. 2) suggested that receipt within 48 months of diagnosis was associated with improved OS, whereas after later receipt showed no significant difference; we therefore incorporated this duration-based window as time-dependent indicator in our models. The following approaches are an approximation and do not fully substitute for a comprehensive time-dependent treatment model.

Despite these limitations, our findings highlight persistent disparities in access to stereotactic radiation for patients with BC BM and the need for strategies to promote equitable implementation of evidence-based guidelines. One key strategy could be to strengthen referral networks between community oncology practices and high-volume tertiary centers to facilitate access to multidisciplinary brain metastasis clinics. Virtual tumor boards and telemedicine consultations could also help extend specialty expertise to community settings. Changes to policy and reimbursement structures may also play an important role in promoting equity in guideline concordant care.

These results also underscore the need for prospective studies evaluating the role of SRT in the context of modern CNS-penetrant systemic therapies. Future trials should investigate optimal integration of radiation and systemic treatment, including whether radiation can be safely deferred in selected patients treated with CNS-penetrating systemic therapies and whether combining SRT with CNS-penetrant therapies improves intracranial outcomes. Importantly, these studies should also evaluate whether disparities in access to advanced radiation technologies influence clinical outcomes in the modern treatment era.

In conclusion, our study demonstrates persistent disparities in treatment patterns for patients with BCBM, particularly in access to SRT. After adjusting for systemic therapy, brain radiation was not independently associated with improved overall survival, emphasizing the critical role of systemic therapy in determining patient outcomes. Prospective clinical trials evaluating SRT integration with modern CNS-penetrant systemic therapies are needed to establish evidence-based treatment standards and ensure equitable access to optimal care for patients with BCBM.

## Supplementary Information

Below is the link to the electronic supplementary material.

Supplementary file1 (JPG 187 KB)

Supplementary file2 (JPG 24 KB)

Supplementary file3 (JPG 66 KB)

Supplementary file4 (DOCX 54 KB)

## Data Availability

Data are available in the National Cancer Database (NCDB) upon request at https://www.facs.org/quality-programs/cancer-programs/national-cancer-database/.
