# Supplementary figures and images for: Radiation treatment patterns for breast cancer brain metastases: an NCDB analysis
Source: Breast Cancer Res Treat. 2026 Apr 22;217(2):26. doi: 10.1007/s10549-026-07955-z (PMC13102853; doi:10.1007/s10549-026-07955-z)

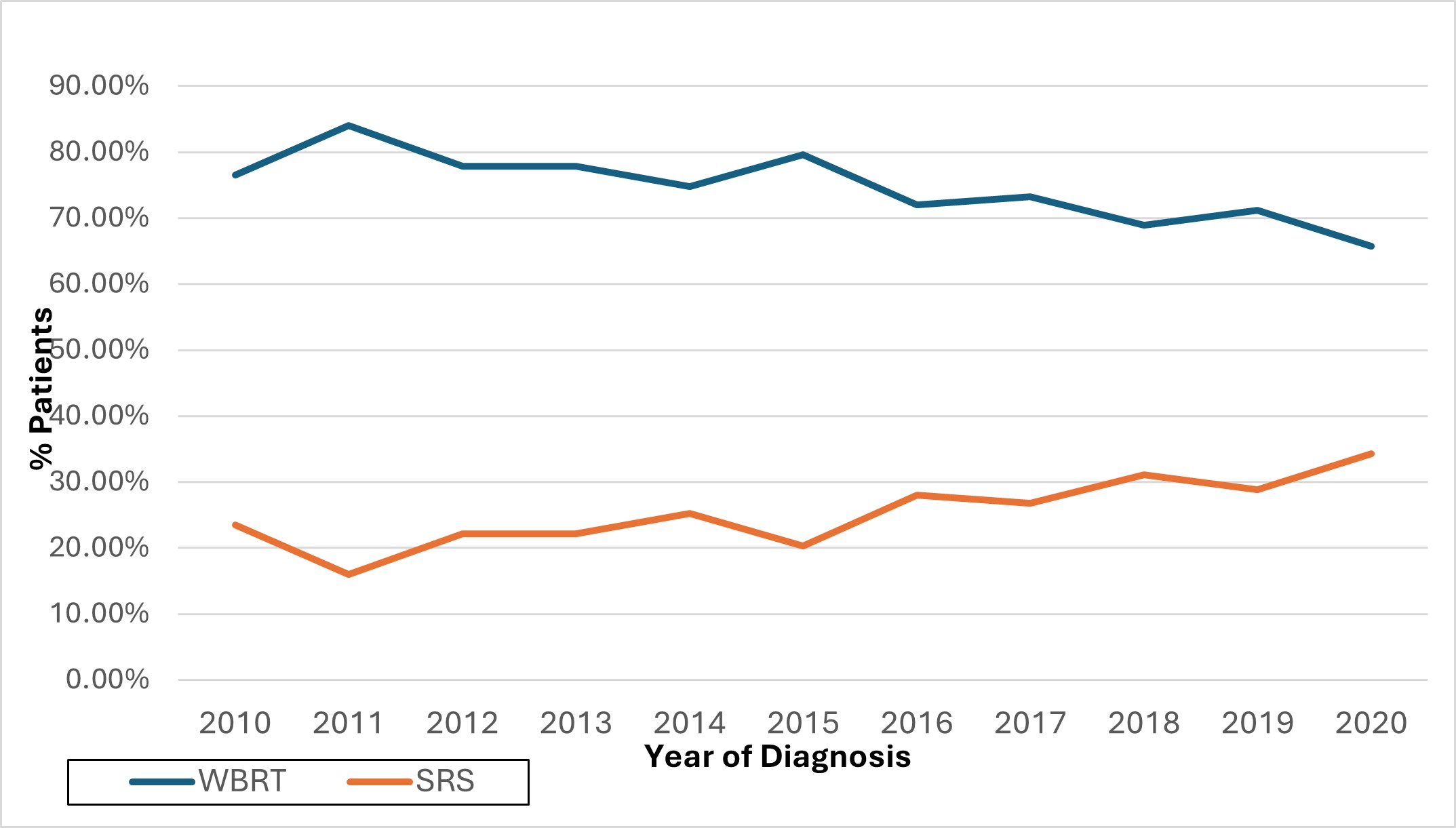

Supplement: Supplementary file 1 — Supplementary file1 (JPG 187 KB) [file 10549_2026_7955_MOESM1_ESM.jpg]

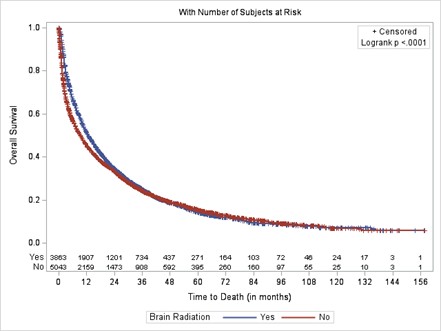

Supplement: Supplementary file 2 — Supplementary file2 (JPG 24 KB) [file 10549_2026_7955_MOESM2_ESM.jpg]

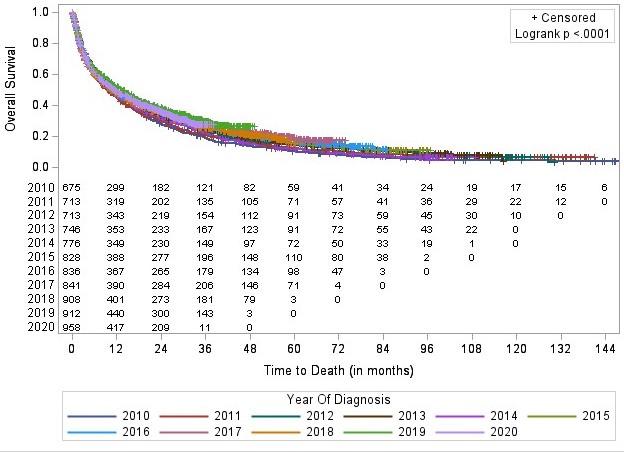

Supplement: Supplementary file 3 — Supplementary file3 (JPG 66 KB) [file 10549_2026_7955_MOESM3_ESM.jpg]
