# Supplementary material for: Radiation treatment patterns for breast cancer brain metastases: an NCDB analysis
Source: Breast Cancer Res Treat. 2026 Apr 22;217(2):26. doi: 10.1007/s10549-026-07955-z (PMC13102853; doi:10.1007/s10549-026-07955-z)
Supplement: Supplementary file 4 — Supplementary file4 (DOCX 54 KB) [file 10549_2026_7955_MOESM4_ESM.docx]

**Supplemental Data:**

Supplemental Figure 1. Distribution of SRS and WBRT cases for treatment of BCBM between 2010 and 2021.

Supplemental Figure 2: Kaplan-Meier Curve Modeling Overall Survival between Patients Receiving Brain RT or no Brain RT

Supplemental Figure 3: Significant difference in OS among patients with BC BM between 2010 and 2021.

Supplemental Table 1. Estimated association between receipt of brain radiation and overall survival.

| **Variable** | **0-48 months** | | **>48 months** | |
| --- | --- | --- | --- | --- |
|  | **Crude HR (95% CI)** | **P-value** | **Crude HR (95% CI)** | **P-value** |
| **Brain Radiation**  Yes  No | 0.89 (0.85-0.93)  Ref | <0.0001 | 1.09 (0.90-1.32)  Ref | 0.3850 |

Supplemental Table 2. Unweighted and weighted results along with standardized differences as validation for use of OPSW method comparing those who received vs not received brain radiation.

| **Supplemental Table 2—Unweighted and weighted results comparing brain radiation to no brain radiation (n=8,909)** | | | | | | |
| --- | --- | --- | --- | --- | --- | --- |
|  | **Unweighted** | | | **Weighted^b^** | | |
|  | **Brain Radiation (n=3,864)** | **No Brain Radiation (n=5,045)** | **Standardize Difference^a^** | **Brain Radiation (n=3,864^)c^** | **No Brain Radiation (n=5,045)^c^** | **Standardize Difference^d^** |
| **Age** |  |  | 0.1505 |  |  | 0^e^ |
| Median (IQR) | 60 (52, 68) | 62 (53, 70) |  | 62 (54, 69) | 61 (54, 69) |  |
|  |  |  |  |  |  |  |
| **Race**, n (%) |  |  |  |  |  |  |
| White | 2946 (76.9%) | 3821 (76.4%) | 0.0110 | 77.7% | 77.7% | 0^e^ |
| African American/Black | 714 (18.6%) | 924 (18.5%) | 0.0040 | 17.9% | 17.9% | 0^e^ |
| Asian | 111 (2.9%) | 163 (3.3%) | 0.0210 | 3.1% | 3.1% | 0^e^ |
| Other | 60 (1.6%) | 91 (1.8%) | 0.0197 | 1.3% | 1.3% | 0^e^ |
|  |  |  |  |  |  |  |
| **Ethnicity,** n (%) |  |  |  |  |  |  |
| Non-Hispanic | 3508 (92.9%) | 4503 (91.9%) | 0.0393 | 92.9% | 92.9% | 0^e^ |
| Hispanic | 268 (7.1%) | 399 (8.1%) | 0.0393 | 7.1% | 7.1% | 0^e^ |
|  |  |  |  |  |  |  |
| **Charlson-Deyo Score**, n (%) |  |  |  |  |  |  |
| 0 | 3118 (80.7%) | 4015 (79.6%) | 0.0278 | 79.2% | 79.2% | 0^e^ |
| 1 | 505 (13.1%) | 679 (13.5%) | 0.0115 | 13.9% | 13.9% | 0^e^ |
| 2 | 152 (3.9%) | 209 (4.1%) | 0.0106 | 4.3% | 4.3% | 0^e^ |
| 3+ | 89 (2.3%) | 142 (2.8%) | 0.0324 | 2.6% | 2.6% | 0^e^ |
|  |  |  |  |  |  |  |
| **Insurance**, n (%) |  |  |  |  |  |  |
| Not Insured | 261 (6.9%) | 381 (7.7%) | 0.0338 | 6.9% | 6.9% | 0^e^ |
| Private Insurance | 1553 (40.8%) | 1741 (35.3%) | 0.1124 | 37.6% | 37.6% | 0^e^ |
| Medicaid | 634 (16.6%) | 784 (15.9%) | 0.0200 | 15.5% | 15.5% | 0^e^ |
| Medicare | 1334 (35.0%) | 1974 (40.1%) | 0.1041 | 39.2% | 39.2% | 0^e^ |
| Other Government | 26 (0.7%) | 47 (1.0%) | 0.0301 | 0.8% | 0.8% | 0^e^ |
|  |  |  |  |  |  |  |
| **Median Income Quartiles**, n (%) |  |  |  |  |  |  |
| <$46,227 | 666 (17.2%) | 847 (16.8%) | 0.0119 | 16.4% | 16.1% | 0.0078 |
| $46,227-$57,856 | 769 (19.9%) | 1037 (20.6%) | 0.0163 | 19.9% | 20.9% | 0.0257 |
| $57,587-$74,062 | 814 (21.1%) | 983 (19.5%) | 0.0393 | 21.3% | 19.0% | 0.0571 |
| $74,063 or higher | 1063 (27.5%) | 1519 (30.1%) | 0.0574 | 28.2% | 30.6% | 0.0532 |
| Unknown | 552 (14.3%) | 659 (13.1%) | 0.0356 | 14.2% | 13.4% | 0.0258 |
|  |  |  |  |  |  |  |
| **Education**, n (%) |  |  |  |  |  |  |
| 15.3% or higher | 799 (20.7%) | 1120 (22.2%) | 0.0371 | 20.6% | 21.6% | 0.0252 |
| 9.1%-15.2% | 1018 (26.3%) | 1275 (25.3%) | 0.0245 | 26.0% | 25.0% | 0.0223 |
| 5.0%-9.0% | 911 (23.6%) | 1218 (24.1%) | 0.0133 | 23.6% | 24.4% | 0.0187 |
| <5.0% | 598 (15.5%) | 782 (15.5%) | 0.0007 | 15.9% | 15.8% | 0.0026 |
| Unknown | 538 (13.9%) | 650 (12.9%) | 0.0305 | 14.0% | 13.2% | 0.0223 |
|  |  |  |  |  |  |  |
| **Community**, n (%) |  |  |  |  |  |  |
| Rural | 62 (1.7%) | 72 (1.5%) | 0.0158 | 1.6% | 1.6% | 0^e^ |
| Urban | 500 (13.4%) | 583 (11.8%) | 0.0463 | 12.6% | 12.6% | 0^e^ |
| Metro | 3176 (85.0%) | 4269 (86.7%) | 0.0497 | 85.8% | 85.8% | 0^e^ |
|  |  |  |  |  |  |  |
| **Nodal**, n (%) |  |  |  |  |  |  |
| 0 | 811 (21.0%) | 1108 (22.0%) | 0.0237 | 21.7% | 21.6% | 0.0013 |
| 1 | 1448 (37.5%) | 1850 (36.7%) | 0.0166 | 36.5% | 37.7% | 0.0267 |
| 2 | 407 (10.5%) | 456 (9.0%) | 0.0503 | 10.4% | 9.3% | 0.0371 |
| 3 | 578 (15.0%) | 582 (11.5%) | 0.1011 | 14.2% | 12.3% | 0.0577 |
| Unknown | 620 (16.0%) | 1049 (20.8%) | 0.1227 | 17.2% | 19.0% | 0.0474 |
|  |  |  |  |  |  |  |
| **Grade**, n (%) |  |  |  |  |  |  |
| 1 | 151 (3.9%) | 223 (4.4%) | 0.0256 | 4.1% | 4.4% | 0.0154 |
| 2 | 781 (20.2%) | 1145 (22.7%) | 0.0605 | 21.0% | 23.2% | 0.0535 |
| 3 | 1381 (35.7%) | 1377 (27.3%) | 0.1825 | 34.0% | 29.0% | 0.1072 |
| Unknown | 1551 (40.1%) | 2300 (45.6%) | 0.1103 | 41.0% | 43.4% | 0.0495 |
|  |  |  |  |  |  |  |
| **Subtype**, n (%) |  |  |  |  |  |  |
| ER+/PR+/HER2- | 1012 (26.2%) | 1635 (32.4%) | 0.1369 | 29.9% | 29.9% | 0^e^ |
| ER+/PR (+ or -)/HER2+ | 571 (14.8%) | 619 (12.3%) | 0.0734 | 13.9% | 13.9% | 0^e^ |
| ER-/PR-/HER2+ | 492 (12.7%) | 446 (8.8%) | 0.1257 | 10.5% | 10.5% | 0^e^ |
| ER-/PR-/HER2- | 893 (23.1%) | 812 (16.1%) | 0.1774 | 19.6% | 19.6% | 0^e^ |
| Unknown | 896 (23.2%) | 1533 (30.4%) | 0.1631 | 26.1% | 26.1% | 0^e^ |
|  |  |  |  |  |  |  |
| **Treatment facility type**, n (%) |  |  |  |  |  |  |
| Community Cancer Program | 239 (6.6%) | 355 (7.5%) | 0.0350 | 6.9% | 6.9% | 0^e^ |
| Comprehensive Community Cancer Program | 1377 (38.1%) | 1787 (37.8%) | 0.0060 | 37.8% | 37.8% | 0^e^ |
| Academic/Research Program | 1259 (34.8%) | 1614 (34.1%) | 0.0145 | 34.4% | 34.4% | 0^e^ |
| Integrated Network Cancer Program | 741 (20.5%) | 973 (20.6%) | 0.0021 | 20.9% | 20.9% | 0^e^ |
|  |  |  |  |  |  |  |
| **Systemic Therapy**, n (%) |  |  |  |  |  |  |
| Yes | 3188 (82.6%) | 3328 (66.1%) | 0.3846 | 76.5% | 76.5% | 0^e^ |
| No | 672 (17.4%) | 1707 (33.9%) | 0.3846 | 23.5% | 23.5% | 0^e^ |

^a^ Absolute difference in means or proportions divided by pooled standard deviation. Imbalance between brain radiation and no brain radiation groups was defined as an absolute value greater than 0.10; smaller values indicated better balance.

^b^ Using overlap weighting to build analytical cohort in which group or treatment type (brain radiation or no brain radiation) is independent of baseline characteristics influencing the initiation of certain treatment

^c^ Overlap-weighted proportions.

^d^ Overlap-weighted standardized differences. Age, race, ethnicity, charlson-deyo score, insurance, community, subtype, facility type and systemic therapy (use of chemotherapy, hormone therapy and/or immunotherapy) were used to estimate the weights.

^e^ Overlapping weights resulted in exact balance for this variable.

Supplemental Table 3. Unweighted and weighted results along with standardized differences as validation for use of OPSW method comparing those who received WBRT vs those who received SRS.

| **Supplemental Table 3—Unweighted comparing WBRT to SRS (n=3,864)** | | | | | | |
| --- | --- | --- | --- | --- | --- | --- |
|  | **Unweighted** | | | **Weighted^b^** | | |
|  | **WBRT**  **(n=2,863)** | **SRS**  **(n=1,001)** | **Standardize Difference^a^** | **WBRT^c^ (n=2,863)** | **SRS^c^**  **(n=1,001)** | **Standardize Difference^d^** |
| **Age** |  |  | 0.0606 |  |  | 0^e^ |
| Median (IQR) | 60 (52, 68) | 59 (51, 67) |  | 61 (53, 69) | 61 (53, 68) |  |
|  |  |  |  |  |  |  |
| **Race**, n (%) |  |  |  |  |  |  |
| White | 2166 (76.3%) | 780 (78.5%) | 0.0533 | 79.0% | 79.0% | 0^e^ |
| African American/Black | 551 (19.4%) | 163 (16.4%) | 0.0783 | 16.7% | 16.7% | 0^e^ |
| Asian | 72 (2.5%) | 39 (3.9%) | 0.0787 | 3.3% | 3.3% | 0^e^ |
| Other | 49 (1.7%) | 11 (1.1%) | 0.0524 | 1.0% | 1.0% | 0^e^ |
|  |  |  |  |  |  |  |
| **Ethnicity,** n (%) |  |  |  |  |  |  |
| Non-Hispanic | 2596 (93.1%) | 912 (92.4%) | 0.0262 | 93.6% | 93.6% | 0^e^ |
| Hispanic | 193 (6.9%) | 75 (7.6%) | 0.0262 | 6.4% | 6.4% | 0^e^ |
|  |  |  |  |  |  |  |
| **Charlson-Deyo Score**, n (%) |  |  |  |  |  |  |
| 0 | 2293 (80.1%) | 825 (82.4%) | 0.0596 | 81.0% | 81.0% | 0^e^ |
| 1 | 391 (13.7%) | 114 (11.4%) | 0.0686 | 12.3% | 12.3% | 0^e^ |
| 2 | 107 (3.7%) | 45 (4.5%) | 0.0382 | 4.6% | 4.6% | 0^e^ |
| 3+ | 72 (2.5%) | 17 (1.7%) | 0.0569 | 2.1% | 2.1% | 0^e^ |
|  |  |  |  |  |  |  |
| **Insurance**, n (%) |  |  |  |  |  |  |
| Not Insured | 207 (7.4%) | 54 (5.4%) | 0.0783 | 5.9% | 5.9% | 0^e^ |
| Private Insurance | 1132 (40.2%) | 421 (42.4%) | 0.0444 | 40.3% | 40.3% | 0^e^ |
| Medicaid | 453 (16.1%) | 181 (18.2%) | 0.0567 | 16.5% | 16.5% | 0^e^ |
| Medicare | 1003 (35.6%) | 331 (33.3%) | 0.0483 | 36.6% | 36.6% | 0^e^ |
| Other Government | 20 (0.7%) | 6 (0.6%) | 0.0131 | 0.6% | 0.6% | 0^e^ |
|  |  |  |  |  |  |  |
| **Median Income Quartiles**, n (%) |  |  |  |  |  |  |
| <$46,227 | 521 (18.2%) | 145 (14.5%) | 0.1005 | 16.6% | 13.4% | 0.0906 |
| $46,227-$57,856 | 589 (20.6%) | 180 (18.0%) | 0.0657 | 19.5% | 18.8% | 0.0191 |
| $57,587-$74,062 | 593 (20.7%) | 221 (22.1%) | 0.0333 | 21.5% | 22.4% | 0.0227 |
| $74,063 or higher | 756 (26.4%) | 307 (30.7%) | 0.0945 | 27.9% | 31.2% | 0.0724 |
| Unknown | 404 (14.1%) | 148 (14.8%) | 0.0192 | 14.4% | 14.1% | 0.0074 |
|  |  |  |  |  |  |  |
| **Education**, n (%) |  |  |  |  |  |  |
| 15.3% or higher | 594 (20.7%) | 205 (20.5%) | 0.0066 | 19.8% | 19.0% | 0.0208 |
| 9.1%-15.2% | 771 (26.9%) | 247 (24.7%) | 0.0515 | 25.7% | 25.1% | 0.0152 |
| 5.0%-9.0% | 692 (24.2%) | 219 (21.9%) | 0.0545 | 24.7% | 23.4% | 0.0314 |
| <5.0% | 411 (14.4%) | 187 (18.7%) | 0.1167 | 15.5% | 18.9% | 0.0887 |
| Unknown | 395 (13.8%) | 143 (14.3%) | 0.0141 | 14.2% | 13.7% | 0.0150 |
|  |  |  |  |  |  |  |
| **Community**, n (%) |  |  |  |  |  |  |
| Rural | 43 (1.6%) | 19 (2.0%) | 0.0318 | 1.8% | 1.8% | 0^e^ |
| Urban | 395 (14.2%) | 105 (10.9%) | 0.1016 | 12.2% | 12.2% | 0^e^ |
| Metro | 2335 (84.2%) | 841 (87.2%) | 0.0842 | 86.0% | 86.0% | 0^e^ |
|  |  |  |  |  |  |  |
| **Nodal**, n (%) |  |  |  |  |  |  |
| 0 | 599 (20.9%) | 212 (21.2%) | 0.0063 | 20.7% | 21.4% | 0.0155 |
| 1 | 1057 (36.9%) | 391 (39.1%) | 0.0441 | 36.9% | 38.5% | 0.0325 |
| 2 | 301 (10.5%) | 106 (10.6%) | 0.0025 | 10.8% | 10.5% | 0.0098 |
| 3 | 421 (14.7%) | 157 (15.7%) | 0.0273 | 15.0% | 15.8% | 0.0238 |
| Unknown | 485 (16.9%) | 135 (13.5%) | 0.0963 | 16.6% | 13.8% | 0.0770 |
|  |  |  |  |  |  |  |
| **Grade**, n (%) |  |  |  |  |  |  |
| 1 | 120 (4.2%) | 31 (3.1%) | 0.0584 | 4.2% | 3.3% | 0.0498 |
| 2 | 577 (20.2%) | 204 (20.4%) | 0.0056 | 20.8% | 20.6% | 0.0047 |
| 3 | 1023 (35.7%) | 358 (35.8%) | 0.0007 | 35.2% | 35.0% | 0.0032 |
| Unknown | 1143 (39.9%) | 408 (40.8%) | 0.0170 | 39.8% | 41.4% | 0.0264 |
|  |  |  |  |  |  |  |
| **Subtype**, n (%) |  |  |  |  |  |  |
| ER+/PR+/HER2- | 724 (25.3%) | 288 (28.8%) | 0.0785 | 28.7% | 28.7% | 0^e^ |
| ER+/PR (+ or -)/HER2+ | 424 (14.8%) | 147 (14.7%) | 0.0035 | 13.6% | 13.6% | 0^e^ |
| ER-/PR-/HER2+ | 365 (12.7%) | 127 (12.7%) | 0.0018 | 12.8% | 12.8% | 0^e^ |
| ER-/PR-/HER2- | 693 (24.2%) | 200 (20.0%) | 0.1020 | 20.7% | 20.7% | 0^e^ |
| Unknown | 657 (22.9%) | 239 (23.9%) | 0.0219 | 24.3% | 24.3% | 0^e^ |
|  |  |  |  |  |  |  |
| **Treatment facility type**, n (%) |  |  |  |  |  |  |
| Community Cancer Program | 196 (7.3%) | 43 (4.6%) | 0.1114 | 5.2% | 5.2% | 0^e^ |
| Comprehensive Community Cancer Program | 1054 (39.2%) | 323 (34.9%) | 0.0881 | 35.7% | 35.7% | 0^e^ |
| Academic/Research Program | 885 (32.9%) | 374 (40.4%) | 0.1571 | 38.7% | 38.7% | 0^e^ |
| Integrated Network Cancer Program | 556 (20.7%) | 185 (20.0%) | 0.0164 | 20.5% | 20.5% | 0^e^ |
|  |  |  |  |  |  |  |
| **Chemotherapy**, n (%) |  |  |  |  |  |  |
| Yes | 1787 (63.5%) | 698 (70.7%) | 0.1545 | 67.4% | 67.4% | 0^e^ |
| No | 1028 (36.5%) | 289 (29.3%) | 0.1545 | 32.6% | 32.6% | 0^e^ |
|  |  |  |  |  |  |  |
| **Hormone therapy**, n (%) |  |  |  |  |  |  |
| Yes | 1082 (38.4%) | 425 (43.2%) | 0.0977 | 42.6% | 42.6% | 0^e^ |
| No | 1733 (61.6%) | 558 (56.8%) | 0.0977 | 57.4% | 57.4% | 0^e^ |
|  |  |  |  |  |  |  |
| **Immunotherapy**, n (%) |  |  |  |  |  |  |
| Yes | 595 (20.9%) | 267 (26.8%) | 0.1377 | 25.1% | 25.1% | 0^e^ |
| No | 2252 (79.1%) | 731 (73.2%) | 0.1377 | 74.9% | 74.9% | 0^e^ |

^a^ Absolute difference in means or proportions divided by pooled standard deviation. Imbalance between WBRT and SRS groups was defined as an absolute value greater than 0.10; smaller values indicated better balance.

^b^ Using overlap weighting to build analytical cohort in which group or treatment type (WBRT or SRS) is independent of baseline characteristics influencing the initiation of certain treatment

^c^ Overlap-weighted proportions.

^d^ Overlap-weighted standardized differences. Age, race, ethnicity, charlson-deyo score, insurance, community, subtype, facility type, chemotherapy, hormone therapy and immunotherapy were used to estimate the weights.

^e^ Overlapping weights resulted in exact balance for this variable.

*Abbreviations*: WBRT=Whole brain radiation therapy; SRS=Stereotactic radiosurgery
